# Interface engineering of mesoporous SnO_2_/TiO_2_ heterojunction with tailored defects for light-motivated gas sensor

**DOI:** 10.1093/nsr/nwag584

**Published:** 2026-09-09

**Authors:** Yidong Zou, Yu Deng, Lingxiao Xue, Junhao Ma, Jichun Li, Tao Bo, Yonghui Deng, Qin Yue

**Affiliations:** School of Materials Science and Engineering, Tongji University, Shanghai 201804, China; Department of Chemistry, State Key Laboratory of Coatings for Advanced Equipment, Shanghai Key Laboratory of Molecular Catalysis and Innovative Materials, iChEM, Fudan University, Shanghai 200433, China; Department of Chemistry, State Key Laboratory of Coatings for Advanced Equipment, Shanghai Key Laboratory of Molecular Catalysis and Innovative Materials, iChEM, Fudan University, Shanghai 200433, China; Department of Chemistry, State Key Laboratory of Coatings for Advanced Equipment, Shanghai Key Laboratory of Molecular Catalysis and Innovative Materials, iChEM, Fudan University, Shanghai 200433, China; Department of Chemistry, State Key Laboratory of Coatings for Advanced Equipment, Shanghai Key Laboratory of Molecular Catalysis and Innovative Materials, iChEM, Fudan University, Shanghai 200433, China; Department of Chemistry, State Key Laboratory of Coatings for Advanced Equipment, Shanghai Key Laboratory of Molecular Catalysis and Innovative Materials, iChEM, Fudan University, Shanghai 200433, China; Ningbo Institute of Materials Technology and Engineering, Chinese Academy of Sciences, Ningbo 315201, China; Department of Chemistry, State Key Laboratory of Coatings for Advanced Equipment, Shanghai Key Laboratory of Molecular Catalysis and Innovative Materials, iChEM, Fudan University, Shanghai 200433, China; Institute of Fundamental and Frontier Sciences, University of Electronic Science and Technology of China, Chengdu 610054, China

**Keywords:** mesoporous material, interface assembly, photoactivation, doping engineering, gas sensor

## Abstract

The convergence of embodied intelligence, neuromorphic computing, and the Internet of Things has propelled intelligent gas sensors to the forefront of artificial olfaction, enabling adaptive, context-aware, and biologically inspired odor perception. Tailoring active sites or defects in sensing materials is an efficient strategy to confer selective gas-fingerprint recognition and for amplifying sensor sensitivity. Herein, a facile ligand-mediated interfacial co-assembly strategy was developed to construct mesoporous crystalline SnO_2_/TiO_2_ composites, which enabled a composition-dependent structural evolution from Sn-substituted TiO_2_ solid solutions to well-defined n–n heterojunctions. Density functional theory calculations reveal that Sn^4+^ ions can be substitutionally incorporated into the TiO_2_ lattice to form charge-balanced Sn–O–Ti bridging units, whereas excessive Sn induces spontaneous phase separation to establish SnO_2_/TiO_2_ heterojunctions. The optimized mesoporous hybrids exhibit ~22-fold higher photocurrent and ~4-fold stronger light-motivated ethanol sensing response than pristine mesoporous TiO_2_ under identical conditions. The outstanding performance was attributed to the site-selective doping that effectively tunes the local electronic structure and promotes the efficient separation and directional transport of photogenerated charges. *In situ* doping allows for the tailoring and engineering of the pore wall chemical microenvironment, affording an active interface conducive to target gas adsorption and detection reactions. This study establishes a design paradigm for coupling surface reactivity regulation with charge transfer engineering in light-activated gas sensors.

## Teaser

Based on a modular doping engineering concept, this study establishes a rational design paradigm for tailoring surface reactivity and charge-transfer kinetics of metal oxides, opening a viable pathway toward light-gated gas sensors.

## INTRODUCTION

Recent advances in the Internet, big data, cloud computing and artificial intelligence are reshaping intelligent sensing and expanding the capabilities of next-generation perception platforms [1–3]. As bioinspired transduction platforms, chemical gas sensors are engineered to emulate the molecular recognition functions of biological olfaction while offering low cost, ease of integration, miniaturization and portability. These sensors enable ultrasensitive detection of trace gases, demonstrating great application potentials in environmental monitoring, smart homes, food safety and non-invasive clinical diagnostics [4–6]. In general, the sensitivity and selectivity of gas sensors are intrinsically governed by the chemical composition, interfacial active-site density and physicochemical properties of sensitive materials, including surface affinity, band structures and active oxygen species [7–10]. Therefore, active sites engineering guided by molecular recognition principles offers a powerful approach to surpass conventional sensitivity limits and achieve reliable multi-analyte selectivity. For inexpensive inorganic metal oxide semiconductors, established sensitization strategies, encompassing heteroatom doping, heterojunction construction and noble metal decoration, leverage lattice modulation, interfacial charge redistribution, and catalytic spillover effects to boost the sensing response, thereby overcoming the intrinsic drawbacks of single-component materials, respectively [4, 11–14].

Advances in nanoscale synthesis and assembly chemistry have enabled metal oxide semiconductors (e.g. WO_3_, In_2_O_3_, SnO_2_, ZnO) with tailored morphologies (e.g. porous architectures, nanosheets, nanospheres) and atomically defined active sites, making them promising sensitive materials for the next-generation gas sensors [15–17]. Rationally engineered sensing platforms based on these materials facilitate real-time, selective detection of trace gases, including H_2_S, CO, NO_2_, ethanol, acetone, formaldehyde, H_2_, CH_4_ and benzene derivatives. Ethanol is an important industrial chemical widely used in medicine, chemical manufacturing and food production. However, exposure to ethanol vapor or its ingestion can produce adverse health effects ranging from respiratory irritation to narcosis and impaired perception. Thus, it is distinctly meaningful to developing efficient ethanol sensors [18].

Among numerous metal oxides, TiO_2_ is particularly attractive as a photoactive material due to its superior stability, scalability and broad utility in various light-harvesting fields [19,20]. Although structural engineering has improved gas-sensing performance, TiO_2_-based sensors still suffer from high operating temperatures, limited sensitivity and sluggish response/recovery dynamics. These limitations mainly arise from inherent electronic and surface-chemical constraints, including insufficient active sites, low oxygen vacancy concentration, weak adsorption affinity for target gases and a wide bandgap (3.0–3.4 eV) [21–23].

Heteroatom doping provides a precise route to modulate local coordination symmetry and electronic states of metal oxides, with dopants adopting substitutional, interstitial, surface-adsorbed or vacancy-coupled configurations depending on mismatches in ionic radius and electronic structure [24–26]. These configurations regulate carrier transport, oxygen-vacancy density, and surface redox kinetics, thereby enabling surface-reactivity optimization for enhanced gas-sensing performance [12,27]. In general, oxygen defects, encompassing oxygen vacancies and interstitial oxygen, can directly influence the gas-sensing performance through the respective facilitative and inhibitory effects of the former and the latter on ion-adsorbed oxygen [28]. Unpaired electrons localized at surface oxygen defects serve as additional reaction sites for adsorbing oxygen and target gas molecules, consequently producing an elevated potential barrier and a thicker depletion layer. A higher concentration of surface oxygen defects leads to a higher sensing sensitivity [29]. For instance, nitrogen incorporation into the anatase TiO_2_ lattice induces lattice distortion, oxygen vacancies and Ti^3+^ defect states, markedly improving acetone sensitivity and selectivity [30]. Additionally, non-metal dopants (e.g. C [31], F [32], P [33]) or transition/post-transition metal dopants (e.g. Cr [34], Ce [35], Cu [36], Co [37], Fe [38]) have been widely explored to tailor TiO_2_-based sensing materials. SnO_2_, a typical *n*-type semiconductor, holds immense promise for advanced gas-sensing platforms owing to its high electron mobility, thermochemical stability and tunable surface/bulk electronic properties [39–42]. SnO_2_ also serves as a UV-active photocatalyst whose deep valence band (VB) endows photogenerated holes with strong oxidizing power for photoelectrocatalytic oxidation, gas sensing and related hole-mediated redox reactions. However, its wide bandgap (*E*_g_ = 3.6 eV) inherently restricts efficient visible-light harvesting, while severe charge-recombination losses further limit its photoactivity [43–45]. Interestingly, as a high-concentration doping strategy, heterojunction construction represents a powerful means of optimizing sensing performance. The formation of heterojunction interfaces fosters synergistic sensitization among multiple components. Concurrently, it reconfigures carrier distribution and surface chemical activity, and modulates the semiconductor band structure and interfacial barriers, thereby substantially enhancing sensing performance [27,46]. In ethanol sensors, the heterojunction architecture promotes rapid reaction with adsorbed oxygen species and consequent electron release, leading to depletion layer narrowing and marked resistance modulation, thereby significantly enhancing the signal response [47]. Thus, the rational engineering of SnO_2_–TiO_2_ heterointerfaces is expected to promote interfacial charge separation for superior photoactivated gas sensing and solar-driven photocurrent.

Guided by modular doping engineering, herein, a ligand-mediated interfacial co-assembly strategy was developed to achieve a precise, site-selective incorporation of Sn^4+^ into mesoporous TiO_2_ framework, generating Sn–O–Ti heterolinkages with tailorable coordination geometry and enhanced charge separation. By varying the Sn concentration (1.0%–30.0%), a series of mesoporous SnO_2_/TiO_2_ composites (mSnO_2_/TiO_2_-*X*) has been prepared, featuring uniform mesopores (28.2–34.3 nm) and high specific surface areas (85.3–116.7 m^2^ g^−1^). Density functional theory (DFT) calculations revealed that low Sn content (mSnO_2_/TiO_2_-5.0) favored isovalent substitution at Ti lattice sites, whereas high Sn content (mSnO_2_/TiO_2_-30.0) drove phase segregation and n–n heterojunction formation. Notably, mSnO_2_/TiO_2_-5.0 delivered a higher photocurrent density under neutral conditions, representing >16 times enhancements over pure mTiO_2_ under identical operational conditions. In addition, mSnO_2_/TiO_2_-30.0 demonstrated 4-fold higher ethanol sensing sensitivity than pure mTiO_2_, featuring rapid response-recovery time (8/29 s) and self-enhancing sensing performance under 365 nm irradiation. The photoactivated sensor presented a novel strategy for advancing high-performance and smart gas sensing platforms.

## RESULTS

Tailored mesoporous SnO_2_/TiO_2_ composites were synthesized through a ligand-mediated interfacial co-assembly strategy (Fig. 1a), wherein amphiphilic poly(ethylene oxide)-*b*-polystyrene (PEO-*b*-PS) diblock copolymer served as a structure-directing agent to precisely govern mesopore architecture. Within a ternary co-solvent medium encompassing tetrahydrofuran (THF), acetic acid and hydrochloric acid, the inorganic precursors [tetrabutyl titanate (TBOT) and SnCl_4_] underwent facile dissolution, subsequently triggering concurrent hydrolysis-condensation cascades. Concurrently, as a non-selective solvent for both PEO and PS domains, THF facilitated the exhaustive micellization of the PEO-*b*-PS copolymer, yielding spatially segregated micellar nanostructures in the continuous phase [48,49]. Furthermore, the hydrolysis of the precursors drove the selective coordination of Ti(IV) and Sn(IV) centers with ether oxygen atoms of the PEO domain in the presence of *p*-hydroxybenzoic acid (PHBA), serving as a molecular bridge. Hydrophilic PHBA molecules contain phenolic hydroxyl (–OH) and carboxyl (–COOH) groups, and these hydroxyl groups associate with hydrophilic PEO segments and coordinate with inorganic precursor oligomers, thus acting as molecular bridges between the PEO chains and oligomers. Moreover, the acidic groups regulate the hydrolysis-condensation kinetics of the TBOT precursor by mitigating rapid evaporation of acetic acid and hydrochloric acid, thereby preserving the assembly driving force. Consequently, phase separation between Ti and Sn precursors during hydrolysis is effectively suppressed. This interaction was underpinned by synergistic electrostatic attraction and coordinate bonding, ultimately affording an integrated organic–inorganic hybrid composite [50]. During programmed evaporation of THF, the spherical organic–inorganic hybrid micelles produced dense packing into mesostructure, which was consolidated via thermal annealing to afford a robust hybrid thin film. Subsequent pyrolysis at 350°C for 3 h under N_2_ atmosphere effected the selective removal of the soft template while concurrently triggering partial carbonization of the polymeric component, yielding *in situ* generated carbon residues. Finally, air calcination removed the carbon residues, yielding novel mesoporous SnO_2_/TiO_2_. Accordingly, Sn-doped composites with graded Sn contents (mSnO_2_/TiO_2_-*X, X* = 1.0%, 3.0%, 5.0%, 10.0%, 20.0% and 30.0%) were synthesized by varying precursor weight ratios.

**Figure 1.**
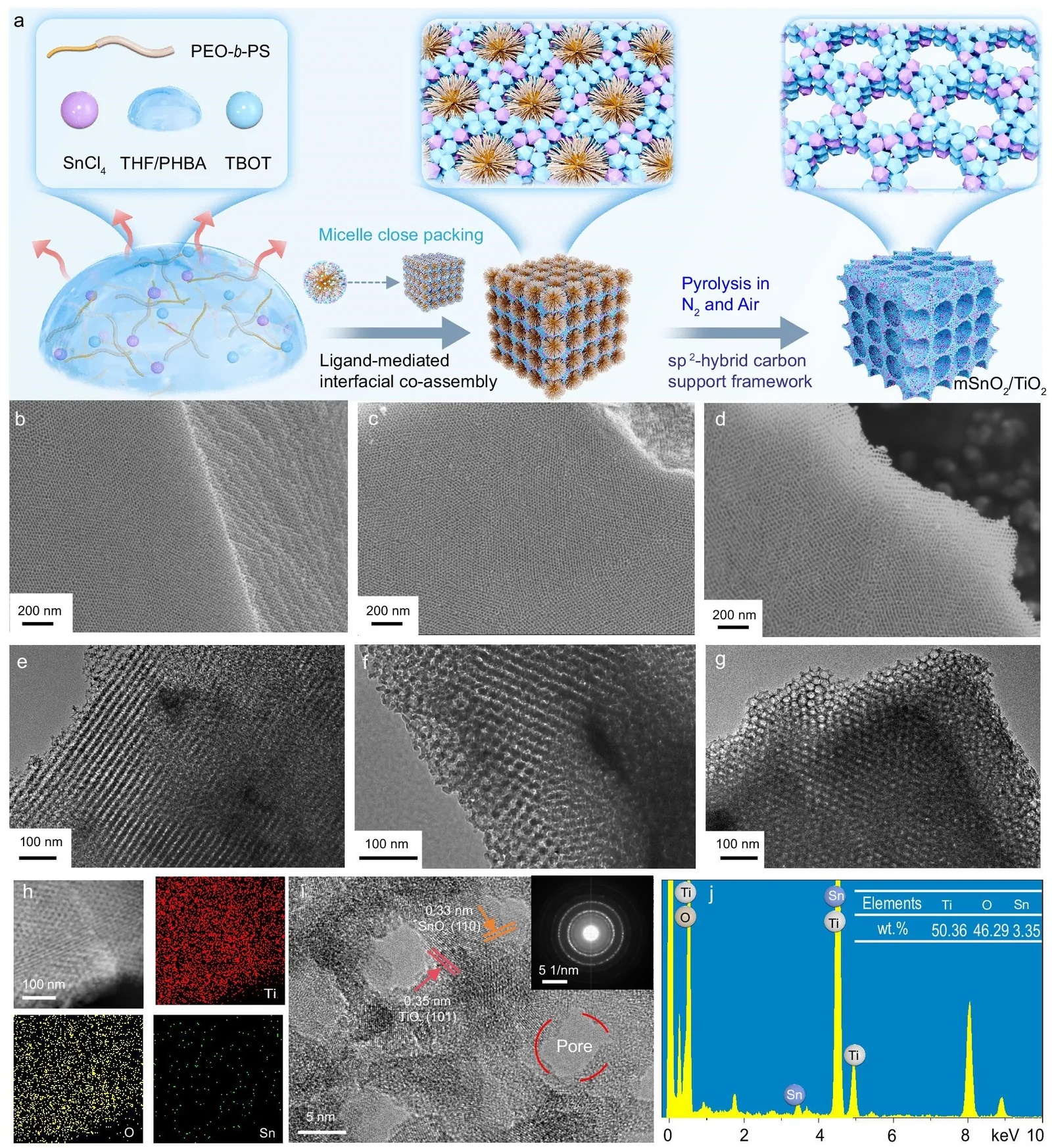
(a) The synthesis route of tailored mesoporous SnO_2_/TiO_2_ composites. (b–g) Morphological characterization of as-prepared mesoporous SnO_2_/TiO_2_ with low Sn^4+^ doping ratios (1–5 wt.%): FESEM and TEM images of mSnO_2_/TiO_2_-1.0 (b, e), mSnO_2_/TiO_2_-3.0 (c, f) and mSnO_2_/TiO_2_-5.0 (d, g), respectively. (h) STEM image and elemental mapping of mSnO_2_/TiO_2_-3.0. (i) HRTEM image of the crystalline mSnO_2_/TiO_2_-3.0 (the inset is the corresponding SAED pattern). (j) The corresponding EDX analysis of mSnO_2_/TiO_2_-3.0.

Field-emission scanning electron microscopy (FESEM) and transmission electron microscopy (TEM) images confirmed that the mSnO_2_/TiO_2_ hybrids with low Sn contents (1.0, 3.0 and 5.0 wt.%) displayed a periodically ordered mesoporous structure featuring spherical pore channels (Fig. 1b–g). Furthermore, ultrasmall SnO_2_ nanoparticles were homogeneously dispersed and anchored onto the pore walls of the mesoporous TiO_2_ framework, concomitant with an appreciable enhancement in crystallinity relative to the pure mesoporous TiO_2_. As the Sn(IV) loading increases from 1.0 to 5.0 wt.%, the long-range mesostructural ordering undergone progressive attenuation, whereas the interconnectivity of the pore network was markedly enhanced. Elemental mapping and high-resolution TEM (HRTEM) analyses of the mSnO_2_/TiO_2_-3.0 sample revealed a homogeneous spatial distribution of Ti, O and Sn elements throughout the hybrid framework, with no discernible particle aggregation or phase domain formation (Fig. 1h). This indicated the absence of discernible phase segregation throughout the synthetic protocol, confirming the homogeneous nanoscale hybridization of TiO_2_ and SnO_2_ domains.

HRTEM image revealed well-defined lattice fringes with interplanar spacings of ~0.33 and 0.35 nm, which were indexed to the {110} plane of SnO_2_ and the {101} plane of TiO_2_, respectively. Concurrently, the mesopore walls were uniformly decorated with ultrafine metal oxide nanoparticles [50,51]. The selected area electron diffraction (SAED) pattern displayed well-defined spotty diffraction rings, confirming the polycrystalline feature of the hybrid material (Fig. 1i). The sharp, well-defined boundaries of the diffraction rings reflect high crystallinity and a fine nanoscale grain structure, suggesting the successful incorporation of dopants without compromising crystalline quality. In addition, the energy-dispersive X-ray spectroscopy (EDX) analysis showed a quantitative Sn content of 3.35 wt.% and closely matched with the nominal feeding stoichiometry, confirming uniform elemental incorporation across the hybrid framework (Fig. 1j). Notably, increasing the Sn^4+^ doping level to 10–30 wt.% enabled the hybrid materials to retain their well-defined mesoporous architecture. The accelerated hydrolysis rate of excessive SnCl_4_ precursor triggered the aggregation of spherical micelles, while the thermodynamically-driven growth of SnO_2_ crystals promoted the pore-wall fusion (Fig. 2a–c). Thus, it led to the emergence of locally columnar pore domains within a globally disordered mesostructure. It indicated that excessive Sn^4+^ incorporation disrupted the condensation kinetics of Ti^4+^ oligomers, thereby accelerating the hydrolysis of titanium precursors. The formation of columnar pores is primarily attributed to the kinetic mismatch between the hydrolysis-crystallization rates of distinct metal salt precursors and the micelle assembly rate. Rapid hydrolysis of SnCl_4_ induces organic–inorganic phase separation, and the imbalance disrupts the local coordination between crystallization and assembly kinetics especially at high Sn-doping levels, driving micelle transformation from spherical to columnar pore geometries [52,53]. Similarly, HRTEM images clearly showed the SnO_2_ {110} and TiO_2_ {101} lattice fringes, and it indicated that the Sn^4+^-rich hybrid material (mSnO_2_/TiO_2_-20.0) retained high crystallinity (Fig. 2d–f). By contrast, the SAED pattern of SnO_2_/TiO_2_-20.0 (inset of Fig. 2e) displayed poorly defined diffraction rings with diffuse boundaries and diminished spot intensity relative to SnO_2_/TiO_2_-5.0, signifying lower crystallinity and larger crystallite sizes. Furthermore, the altered positions of the main diffraction rings point to compositional differences between the two samples. Such high-concentration doping gives rise to a distinct SnO_2_ crystal phase. In addition, small-angle X-ray scattering (SAXS) patterns of all samples exhibited three typical scattering peaks, which were assigned to the 100, 110 and 200 reflections of the mesostructure [54]. Interestingly, the *q*-value of the low-concentration Sn-doped samples was smaller and closer to that of the pure mTiO_2_ (Fig. 3a). In contrast, the *q*-value of the high-concentration Sn-doped samples shifted to larger values, suggesting disruption of order structure, potentially leading to short-range ordering or local structural disorder, which was consistent with the SEM image.

**Figure 2.**
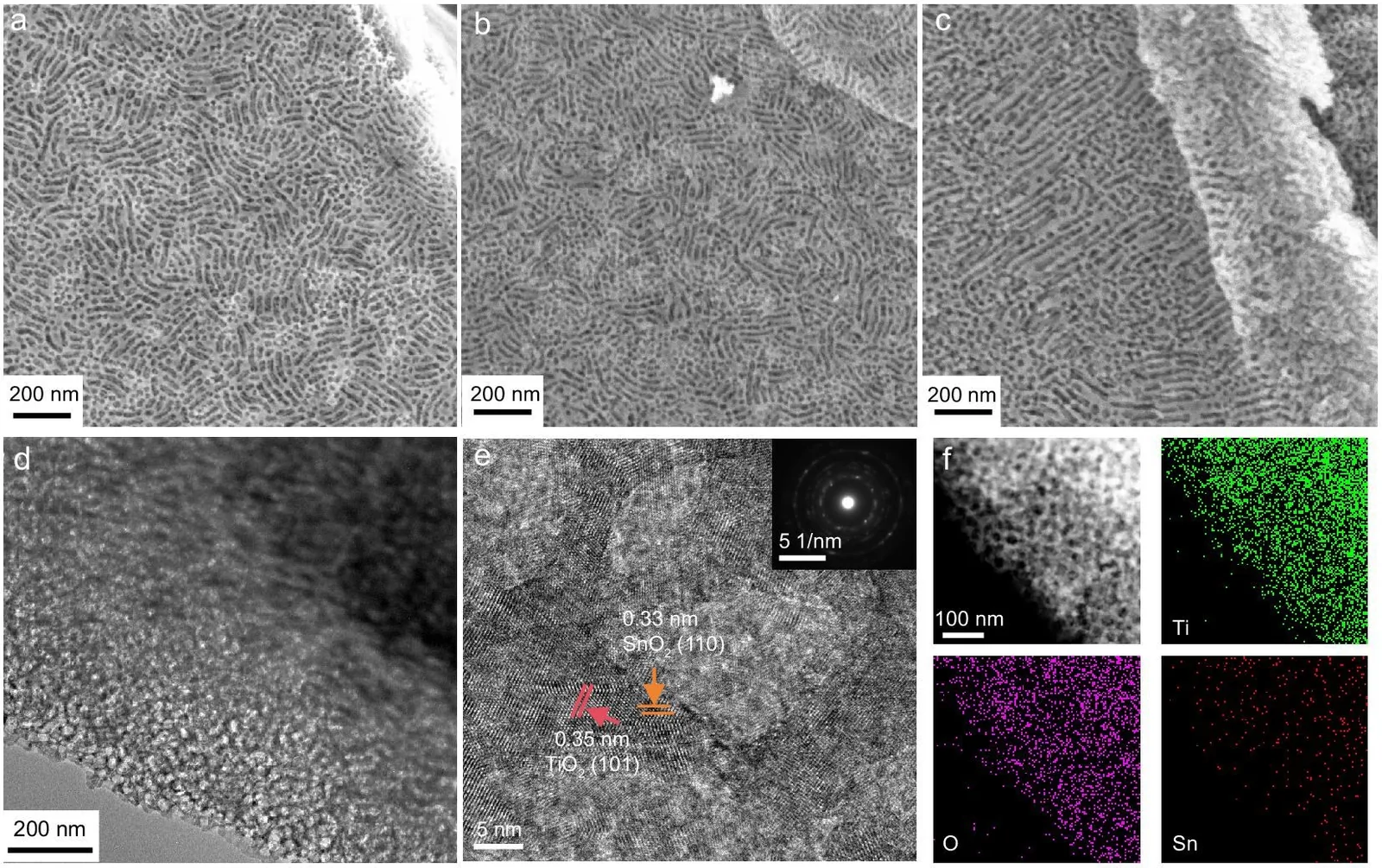
Morphological characterization of tailored mesoporous SnO_2_/TiO_2_ with high Sn^4+^ doping ratios (10–30 wt.%). FESEM images of (a) mSnO_2_/TiO_2_-10.0, (b) mSnO_2_/TiO_2_-20.0 and (c) mSnO_2_/TiO_2_-30.0, respectively. (d) TEM image of mSnO_2_/TiO_2_-20.0. (e) HRTEM image of mSnO_2_/TiO_2_-20.0 (the inset is corresponding SAED pattern). (f) STEM image and elemental mapping of mSnO_2_/TiO_2_-20.0.

**Figure 3.**
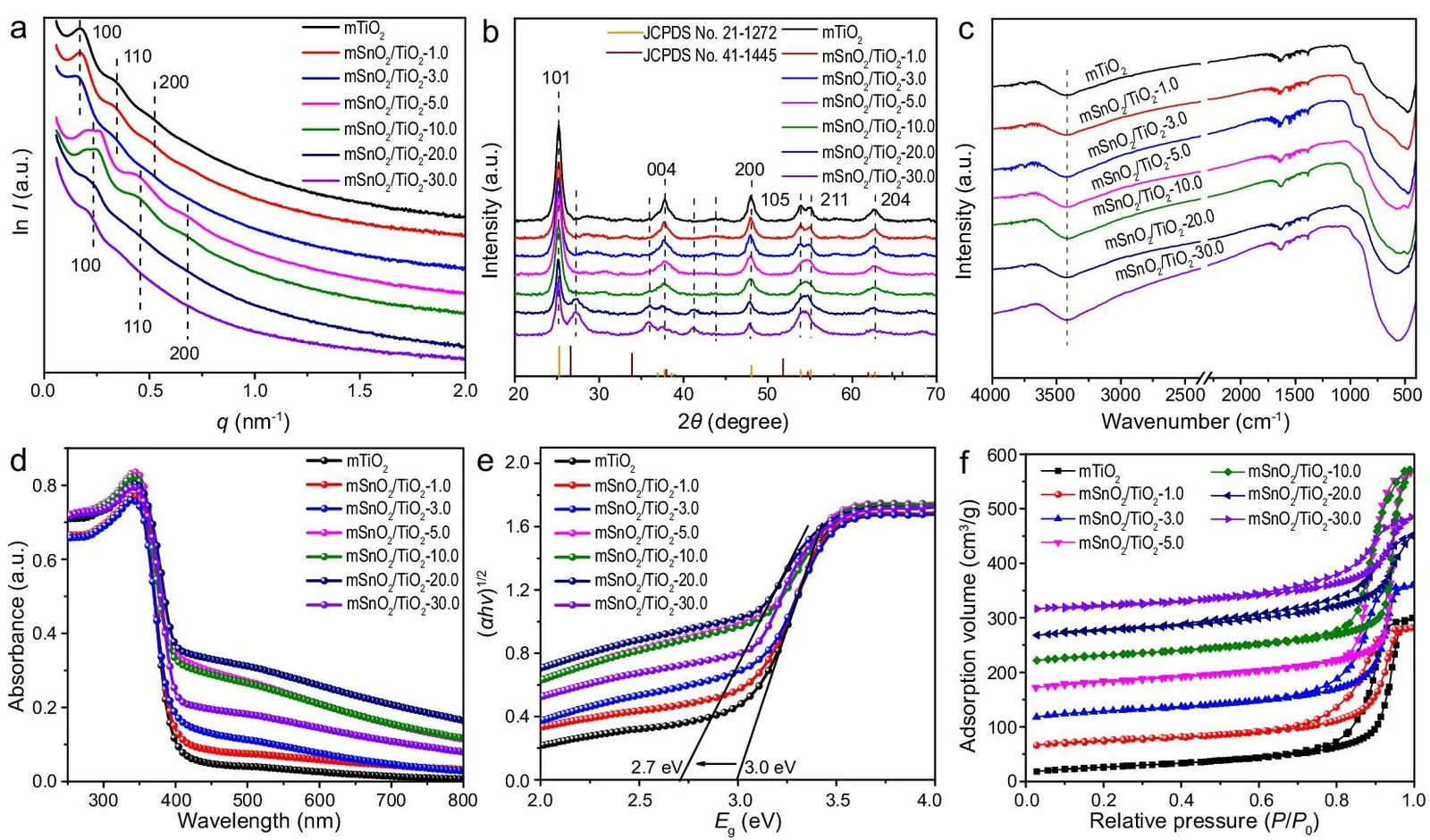
Structural characterization of mSnO_2_/TiO_2_-*X* (*X* = 1.0, 3.0, 5.0, 10.0, 20.0 and 30.0): (a) SAXS patterns, (b) XRD patterns, (c) FT-IR spectra, (d) UV-DRS spectra and (e) Kubelka-Munk function curve plotted against photon energy. (f) Nitrogen adsorption-desorption isotherms (shift upward by 50, 100, 150, 200, 250 and 300 cm^3^ g^−1^, respectively).

In the X-ray diffraction (XRD) spectra, all samples exhibited similar diffraction peaks, and the most prominent diffraction peaks were indexed to the (101), (004) and (200) reflections of anatase TiO_2_ (JCPDS No. 21-1272), respectively (Fig. 3b) [21]. When the Sn doping concentration was low (<5 wt.%), no discernible SnO_2_ diffraction peaks were detected in the XRD pattern of the hybrid material. The peak positions and profiles remained virtually identical to those of pure mTiO_2_, suggesting that Sn^4+^ was incorporated primarily via ionic doping without significantly perturbing the TiO_2_ crystal structure. When the Sn^4+^ content was increased to 5% and 10%, significant broadening of the diffraction peak near 55° was observed, and the peak at ~63° gradually evolved into a broad hump. It suggested that excess Sn^4+^ interfered with the hydrolysis and condensation of titanium precursors during synthesis, leading to reduced crystallinity of the hybrid material, as confirmed by increased full width at half maximum (FWHM) of the (101) anatase reflection. When the Sn^4+^ content exceeded 10 wt.%, a new diffraction peak (~27°) indexed to rutile SnO_2_ (110) reflection emerged in the XRD pattern, confirming the formation of crystalline SnO_2_ phase (JCPDS No. 41–1445) [39–41]. In addition, Fourier transform infrared (FT-IR) spectra of mSnO_2_/TiO_2_ and mTiO_2_ are nearly identical across most spectral regions, except for pronounced differences in the 500–1200 cm^−1^ range, suggesting SnO_2_-related vibrational modes (such as Sn–O) or interfacial Ti–O–Sn bonding (Fig. 3c). Moreover, as the Sn^4+^ content increases, the FT-IR absorption band near 500 cm^−1^ shifted progressively to higher wavenumbers, suggesting local structural distortion and perturbations in the Sn–O and Ti–O vibrational environments [22]. Solid-state UV–Vis diffuse reflectance spectroscopy was employed to probe the optical absorption characteristics of the hybrid materials, providing initial insight into their light-harvesting capability. It revealed that with increasing Sn^4+^ content, the absorbance of the hybrid material in the visible region first increased and then decreased (Fig. 3d). It was ascribed to the fact that Sn^4+^ incorporated substitutionally into the lattice, engendering localized defect states within the band gap that bolstered visible-light absorption under low doping levels. At excessive loading, Sn^4+^ precipitated phase separation, degrading lattice integrity and enhancing light scattering at the expense of absorption, consequently suppressing visible-light absorption [55,56]. Among the tested samples, mSnO_2_/TiO_2_-5.0, mSnO_2_/TiO_2_-10.0 and mSnO_2_/TiO_2_-20.0 exhibited excellent light-harvesting capacity, correlating with a band gap narrowing from 3.0 to 2.7 eV (Fig. 3e). The red-shifted absorption and narrowed band gap indicated that Sn^4+^ incorporation effectively enhanced the material’s light-harvesting capability and modulated its electronic band structure, thereby expanding its applicability in photocatalysis and light-driven gas sensing.

Nitrogen adsorption-desorption isotherms for all samples exhibited typical type-IV curves with an H2-type hysteresis loop, and a sharp capillary condensation step was observed in the relative pressure range of 0.9–0.95 (Fig. 3f), and it implied that all samples exhibited a well-defined mesoporous structure [48]. Interestingly, when the doping concentration was lower than 20 wt.%, the hysteresis loops showed similar widening. In contrast, at higher doping levels (20 and 30 wt.%), the loops became significantly broader, indicating substantial changes in pore structure. The pore size distribution derived from the adsorption branch showed uniform pore sizes, and the average pore size could be adjusted from 28.2 to 34.3 nm (Fig. S1). In addition, the Brunauer–Emmett–Teller (BET) surface areas and total pore volumes of mSnO_2_/TiO_2_ ranged from 85.33 to 116.71 m^2^ g^−1^ and from 0.29 to 0.64 cm^3^ g^−1^, respectively (Table S1), which first increased and then decreased with increasing Sn^4+^ doping concentration. It indicated that excessive Sn^4+^ doping reduced the specific surface area, owing to the transformation of uniform spherical pores into interconnected worm-like pores.

To compare the valence states and bonding forms of hybrid materials with different Sn^4+^ doping amounts, X-ray photoelectron spectra (XPS) analysis was performed on three samples (Fig. S2). The narrow-scan Ti 2p spectra revealed characteristic Ti 2p_3/2_ and Ti 2p_1/2_ double peaks in all samples (Fig. 4a–c). For mSnO_2_/TiO_2_-5.0, the Ti 2p_3/2_ peak consisted of a main component at 458.9 eV and a new shoulder at 456.9 eV, assignable to Ti^4+^ and Ti^3+^, respectively (Fig. 4b) [50,54]. However, the new peak at 456.9 eV vanished in mSnO_2_/TiO_2_-30.0, suggesting that higher Sn^4+^ doping suppressed the formation of Ti^3+^ and altered the Ti coordination environment (Fig. 4c). However, when the Sn^4+^ doping level was relatively low (5.0 wt.%), Sn^4+^ primarily occupied Ti^4+^ lattice sites, inducing local charge redistribution and partial reduction of Ti^4+^ to Ti^3+^. It resulted in a shoulder at ~456.9 eV in the Ti 2p spectrum, in addition to the main Ti^4+^ peak at ~458.9 eV. When the Sn^4+^ content was relatively high (30.0 wt.%), SnO_2_ and TiO_2_ produced phase separation, forming a SnO_2_/TiO_2_ heterojunction rather than a homogeneous solid-solution phase. In this case, the TiO_2_ domains retained structural and electronic integrity, resulting in a Ti 2p spectrum analogous to that observed for pristine mTiO_2_, and some local variations primarily originate from trace Ti^3+^ species, generated by charge transfer across the heterojunction. Similarly, the O 1s spectra confirmed this distinction, these materials resolved into three well-defined deconvoluted peaks assigned to lattice oxygen (*O*_latt_), defective oxygen (*O_v_*) and adsorbed molecular oxygen (*O*_abs_), whereas mSnO_2_/TiO_2_-5.0 showed extra peak contribution for lattice oxygen (Fig. 4d–f and Table S2). It was ascribed to interfacial oxygen species (e.g. Ti–O–Sn bridges) coupled with the SnO_2_-mediated generation of surface oxygen vacancies [55–58]. By contrast, quantitative integral area analysis reveals a pronounced increase in defect oxygen content from 25.5% (mTiO_2_) to 51.2% (mSnO_2_/TiO_2_-30.0) upon Sn doping, establishing controlled defect engineering for the further optimization of sensing performance.

**Figure 4.**
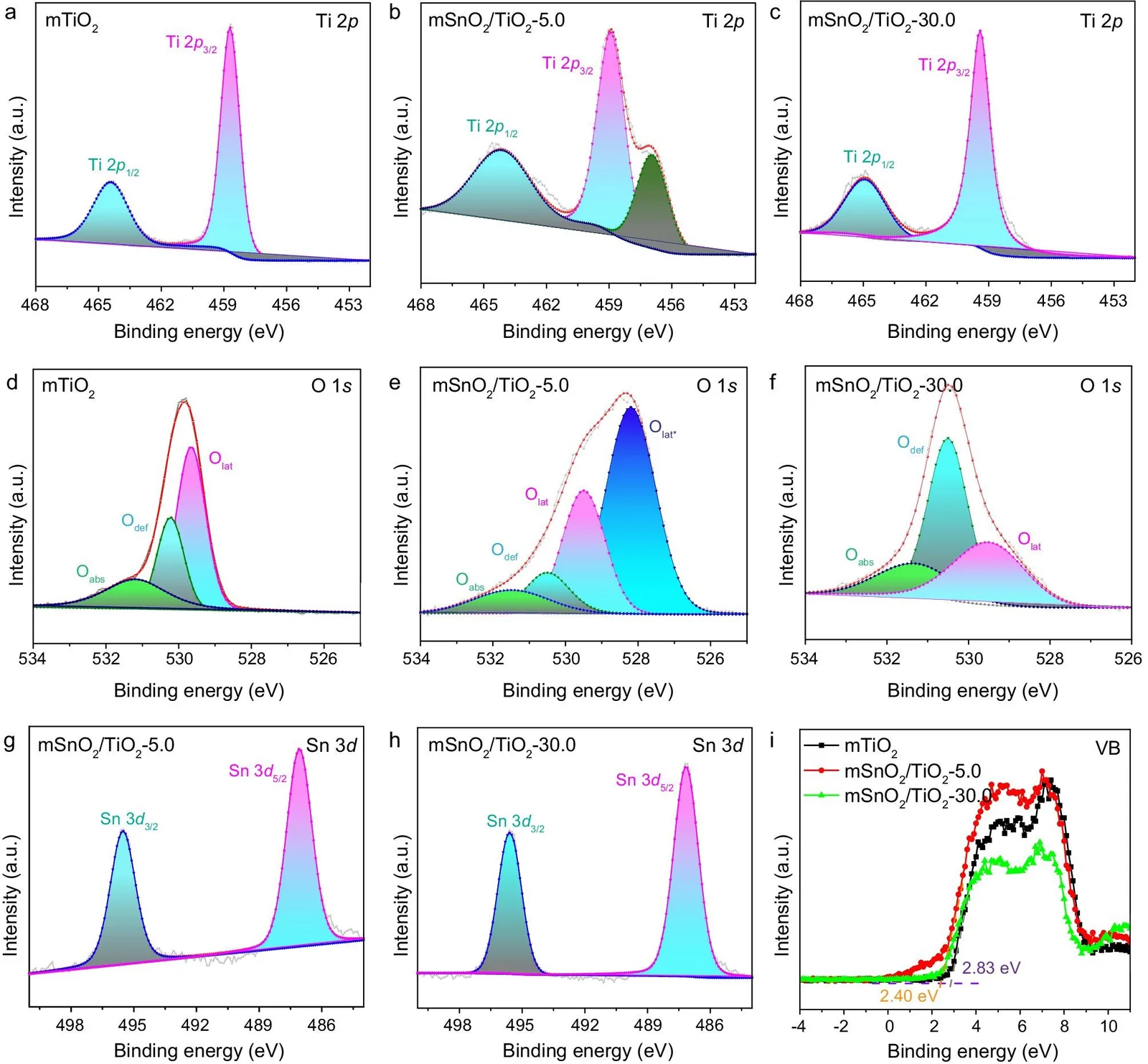
The valence structure of mSnO_2_/TiO_2_-*X* (*X* = 5.0 and 30.0) hybrid materials. Ti 2p spectra of mTiO_2_ (a), mSnO_2_/TiO_2_-5.0 (b) and mSnO_2_/TiO_2_-30.0 (c). O 1s spectra of mTiO_2_ (d), mSnO_2_/TiO_2_-5.0 (e) and mSnO_2_/TiO_2_-30.0 (f). Sn 3d spectra of mSnO_2_/TiO_2_-5.0 (g) and mSnO_2_/TiO_2_-30.0 (h). (i) Valence band distribution XPS spectra.

Interestingly, the Sn 3d spectra of mSnO_2_/TiO_2_-5.0 and mSnO_2_/TiO_2_-30.0 exhibited distinct Sn(IV)-related compositional and interfacial electronic signatures, as evidenced by subtle yet discernible variations in peak profiles, relative intensities and binding energy positions. In mSnO_2_/TiO_2_-5.0, the Sn 3d region (485–500 eV) was dominated by the Sn^4+^ doublet (Sn 3d_5/2_ at ~487.0 eV and Sn 3d_3/2_ at ~495.5 eV), which was consistent with the feature of Sn(IV) coordination environment (Fig. 4g) [50]. In contrast, the Sn 3d spectrum of mSnO_2_/TiO_2_-30.0 exhibited similar Sn^4+^ doublet (Sn 3d_5/2_ at ~487.2 eV and Sn 3d_3/2_ at ~495.6 eV), indicating the typical structure of SnO_2_ species (Fig. 4h). XPS VB spectra revealed that mTiO_2_ exhibited a VB edge at 2.83 eV, whereas mSnO_2_/TiO_2_-5.0 showed a VB edge at 2.40 eV, indicating a 0.43 eV upward shift of the VB edge in mTiO_2_ relative to mSnO_2_/TiO_2_-5.0 (Fig. 4i). It is primarily attributed to the introduction of Sn-related impurity states within the TiO₂ band gap, localized near the valence band maximum. Such states effectively reduce the energy separation between the valence and conduction bands. Furthermore, Sn doping promotes oxygen vacancy formation, and the associated defect states intrude into the band gap, additionally lowering the energy barrier for electron excitation [24,25]. The Sn^4+^-induced valence band offset reflected a fundamental modulation of the electronic structure, and the resulting staggered band alignment enhanced spatial separation of photogenerated carriers and facilitated thermodynamically favorable electron transfer from the SnO_2_-rich phase to TiO_2_, thereby improving charge extraction efficiency.

To elucidate the role of Sn^4+^ incorporation in TiO_2_, Hubbard-U-corrected density functional theory (DFT + *U*) calculations were performed to analyze the structural and electronic properties of mSnO_2_/TiO_2_ hybrid composites. The unit cell of TiO_2_ was optimized, and band structure calculations were performed using the GGA + *U* method with *U* values of 3, 4, 5, 6, 7, 8, 8.5, 9, 9.5 and 10 eV (Table S3) [59–61]. At *U* = 8.5 eV, the calculated lattice parameters of TiO_2_ showed the best agreement with experimental XRD data, and the optimized unit cell parameters were *a* = *b* = 3.992 Å, *c* = 9.796 Å (Fig. S3). The SnO_2_ unit cell was optimized using the generalized gradient approximation with the Perdew–Burke–Ernzerhof functional (i.e. without Hubbard U correction), and the optimized lattice parameters are *a* = *b* = 4.829 Å, *c* = 3.238 Å (space group: *P*_42_/mnm) (Fig. S4) [62]. Based on this model, comparative analyses of the electronic properties were conducted for the mSnO_2_/TiO_2_-5.0 and mSnO_2_/TiO_2_-30.0 heterojunctions (Fig. 5a–f). The results showed that the formation energies for two common Sn^4+^ doping configurations, interstitial incorporation and substitution at the Ti-site in the TiO_2_ lattice were calculated at a low Sn^4+^ doping concentration (e.g. 5.0%) (Fig. S5). In addition, Sn^4+^ incorporation significantly modified the band structure, and total energy calculations showed that the pristine TiO_2_ supercell was more stable than the interstitially doped Sn^4+^ configuration. The total energy of the pristine 3 × 3 × 1 TiO_2_ supercell (band gap = 3.107 eV) was −703.389 eV (Fig. 5a), whereas that of the Sn^4+^-interstitial-doped supercell was −703.237 eV (Fig. 5c). It should be noted that a lower (more negative) total energy indicated greater thermodynamic stability, and the total energy of the Sn^4+^-substituted TiO_2_ supercell (Sn^4+^ substituting for Ti^4+^) was −704.341 eV (Fig. 5d). For a different Ti substitution configuration, the total energy was −704.355 eV (Fig. 5e). Both formation energies were significantly lower than that of the Sn^4+^-interstitial configuration (-703.237 eV), indicating that Ti substitution was thermodynamically favored at low Sn^4+^ concentrations.

**Figure 5.**
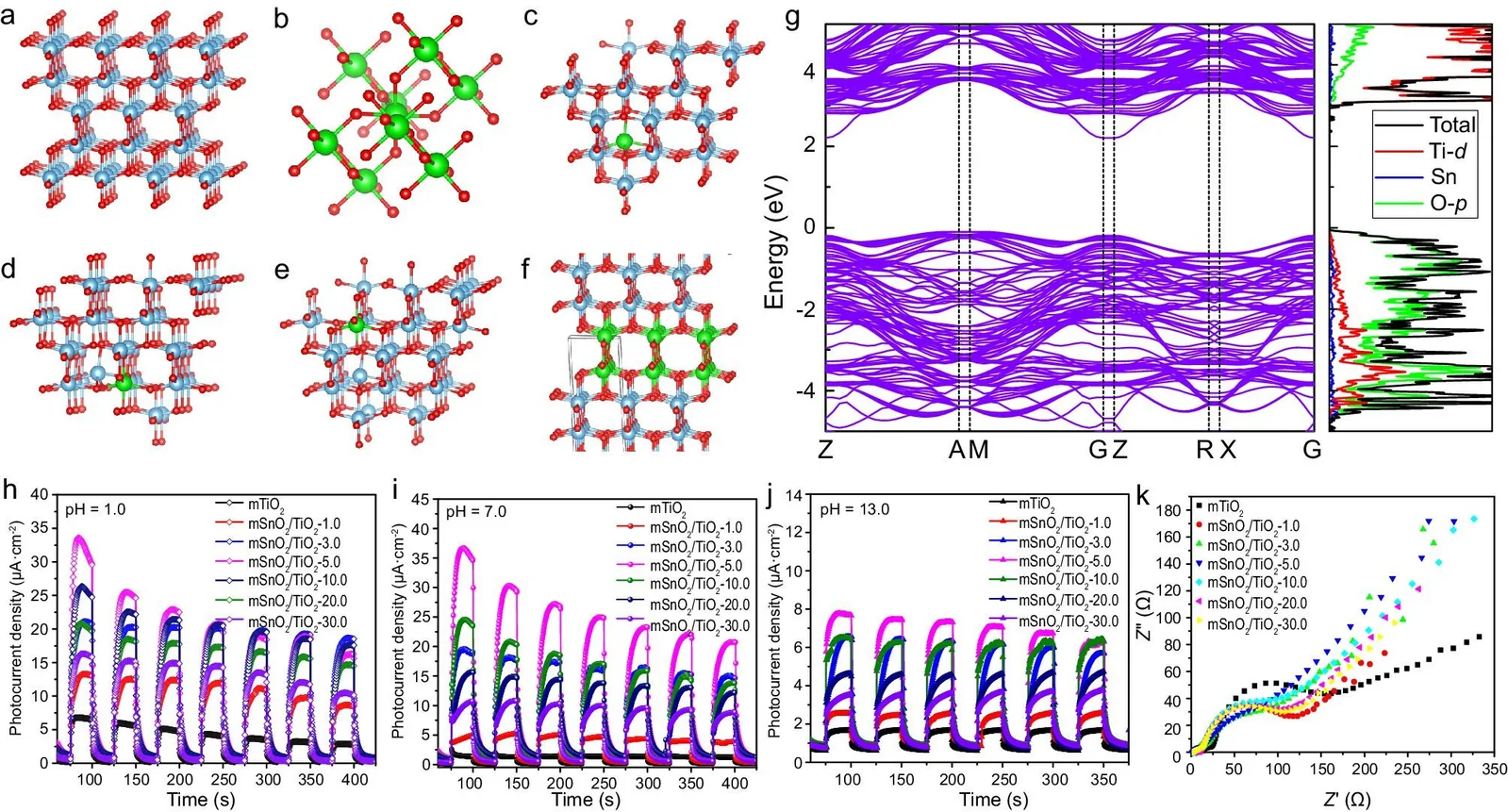
DFT study of the Sn^4+^ doping mechanism in TiO_2_ (red-O atom, blue-Ti atom, green-Sn atom). (a) Optimized TiO_2_ crystal structure. (b) Optimized SnO_2_ crystal structure. Optimized mSnO_2_/TiO_2_-5.0 crystal structure of (c) interstitial lattice, (d) atomic substitution of site I and (e) atomic substitution of site II. (f) Optimized mSnO_2_/TiO_2_-30.0 crystal structure of heterojunction. (g) Energy band structure of mSnO_2_/TiO_2_-30.0. Transient photocurrent density versus time at various pH conditions: (h) pH = 1.0; (i) pH = 7.0 and (j) pH = 13.0. (k) Nyquist plots comparison.

At a relatively high Sn^4+^ doping level (e.g. 30.0 wt.%), an n–n heterojunction comprising SnO_2_ and TiO_2_ phases was constructed based on the expectation of phase segregation (Fig. 5f). The interfacial binding energy calculated as the energy difference between the heterojunction and its separated SnO_2_ and TiO_2_ components confirmed that this configuration was more stable than substitutional or interstitial doping forms. Among all modeled structures, the SnO_2_/TiO_2_ heterojunction exhibited the lowest total energy, indicating its thermodynamic favorability for formation under high Sn^4+^ doping conditions. This structure provided abundant interfacial active sites, which enhanced charge separation and surface reactivity. Density of states and band structure analysis further supported the formation of an n–n heterojunction (Fig. 5g). The SnO_2_/TiO_2_ composite exhibited a narrower band gap, which enhanced visible-light absorption and improved performance in photocatalysis and gas sensing. Subsequently, both vacancy-filling and substitutional doping configurations were calculated, and three distinct scenarios were considered: (i) all six Sn^4+^ ions occupying oxygen vacancies, (ii) all six Sn^4+^ ions substituting Ti^4+^ sites and (iii) three Sn^4+^ ions filling oxygen vacancies while the other three substitute Ti^4+^ sites. Based on these, 15 distinct doping configurations were enumerated (Table S4). In contrast to substitution of Ti^4+^ by smaller cations, Sn^4+^ substitution of Ti^4+^ yielded a more stable lattice structure. However, the structural stability of both Sn^4+^ substitution and vacancy-filling configurations was lower than the SnO_2_/TiO_2_ heterojunction, and thus heterojunction formation was thermodynamically favored at 30 wt.% Sn doping (Fig. S6).

To probe the light-harvesting behavior, photocurrent transients of tailored mSnO_2_/TiO_2_ hybrids were recorded under standard illumination (500 W Xe arc lamp) on a CHI 760E electrochemical workstation. The hybrid materials were fabricated into working electrodes, with photocurrent performance comprehensively probed across acidic (pH 1.0), neutral (pH 7.0) and alkaline (pH 13.0) media. The photocurrent behavior and stability of all samples were interrogated under intermittent on–off illumination. In acidic electrolyte, the mSnO_2_/TiO_2_ hybrids demonstrated substantially enhanced photocurrent densities compared to pure mTiO_2_, and the mSnO_2_/TiO_2_-5.0 registered the maximal average photocurrent density of 20 μA cm^−2^, whereas the remaining hybrid materials maintained values above 10 μA cm^−2^ (Fig. 5h). Nevertheless, these metal oxides engendered progressive photocurrent attenuation during cyclic operation, attributable to corrosive dissolution or surface reconstruction in acidic media. Particularly under acidic conditions, protons congregate at the metal oxide surface, engendering localized acid etching. This substantially reduces the density of active sites, thereby attenuating the photoresponse after prolonged cycling. Notably, after seven light-on/light-off cycles, the photocurrent densities of all hybrid materials remained within the range of 10–20 μA cm^−2^, 4–6 times higher than mTiO_2_ under identical conditions. Under neutral pH conditions, most samples exhibited weaker photocurrent performance compared with acidic environments. However, mSnO_2_/TiO_2_-5.0 showed a significantly enhanced response, averaging up to 25 μA cm^−2^, 16–22 times higher than mTiO_2_ under identical conditions (Fig. 5i). It indicated that low-concentration Sn^4+^ doping could significantly improve the electronic structure and light-harvesting capability of TiO_2_, thereby enhancing its photocurrent response under illumination. Consistently, in alkaline media, mSnO_2_/TiO_2_-5.0 exhibited the highest photocurrent response, four times higher than that of mTiO_2_ under identical conditions (Fig. 5j). However, the overall photocurrent response of the hybrid material significantly decreased, likely resulting from alkaline-induced weakening of binder-electrode adhesion and subsequent local delamination of the active layer. Sn^4+^ doping enhanced the light absorption capability of mTiO_2_ and showed high promise for photocatalytic and solar energy conversion applications. In addition, the EIS impedance spectra revealed that Sn^4+^-doped samples exhibited lower charge-transfer resistances than pure TiO_2_, indicative of an enhanced electron-hole separation and an accelerated electron transport (Fig. 5k). It revealed substantially enhanced interfacial charge-transfer kinetics in the doped samples, which originated from the synergistic effects of Sn^4+^ incorporation. On the one hand, doping engineering increases the surface density of electronic states, and it strengthens interfacial electron coupling, diminishes the activation energy barrier for charge transfer, concomitantly reducing charge-transfer resistance. On the other hand, Sn^4+^ doping induces lattice strain or oxygen vacancy formation, and it fosters efficient photogenerated electron-hole separation while mitigating charge-carrier recombination [63,64].

Furthermore, SnO_2_ was widely employed as an active material in gas sensing due to its excellent sensitivity and fast response/recovery kinetics, whereas pure TiO_2_ exhibited considerably lower intrinsic gas sensing activity and high resistance. Thus, in the hybrid materials, SnO_2_ served as the core sensing material, and samples with high Sn^4+^ content (mSnO_2_/TiO_2_-30.0) were primarily evaluated by microelectron mechanical system (MEMS) sensor (Fig. 6a). Given the high resistance characteristic of the material, the voltage signal was chosen as the measured sensing signal (Fig. 6b). It showed that mSnO_2_/TiO_2_-30.0 exhibited excellent sensing performance toward 50 ppm ethanol with an optimal operating temperature of 280°C, which was attributed to the competition equilibrium between the increase of surface reaction rates and the decrease of active sites (Fig. 6c) [41,42]. Specifically, the sensing tests of mSnO_2_/TiO_2_-30.0 to 50 ppm of common interfering gases showed high selectivity toward ethanol at the same condition (Fig. 6d). In addition, it exhibited typical response–concentration positive correlation and recovery cycle (Fig. 6e and f), indicating its good surface adsorption/desorption kinetics behavior. Interestingly, the sensing response showed a self-enhancement phenomenon, which was possibly attributed to elevated material activity induced by optical or thermal excitation.

**Figure 6.**
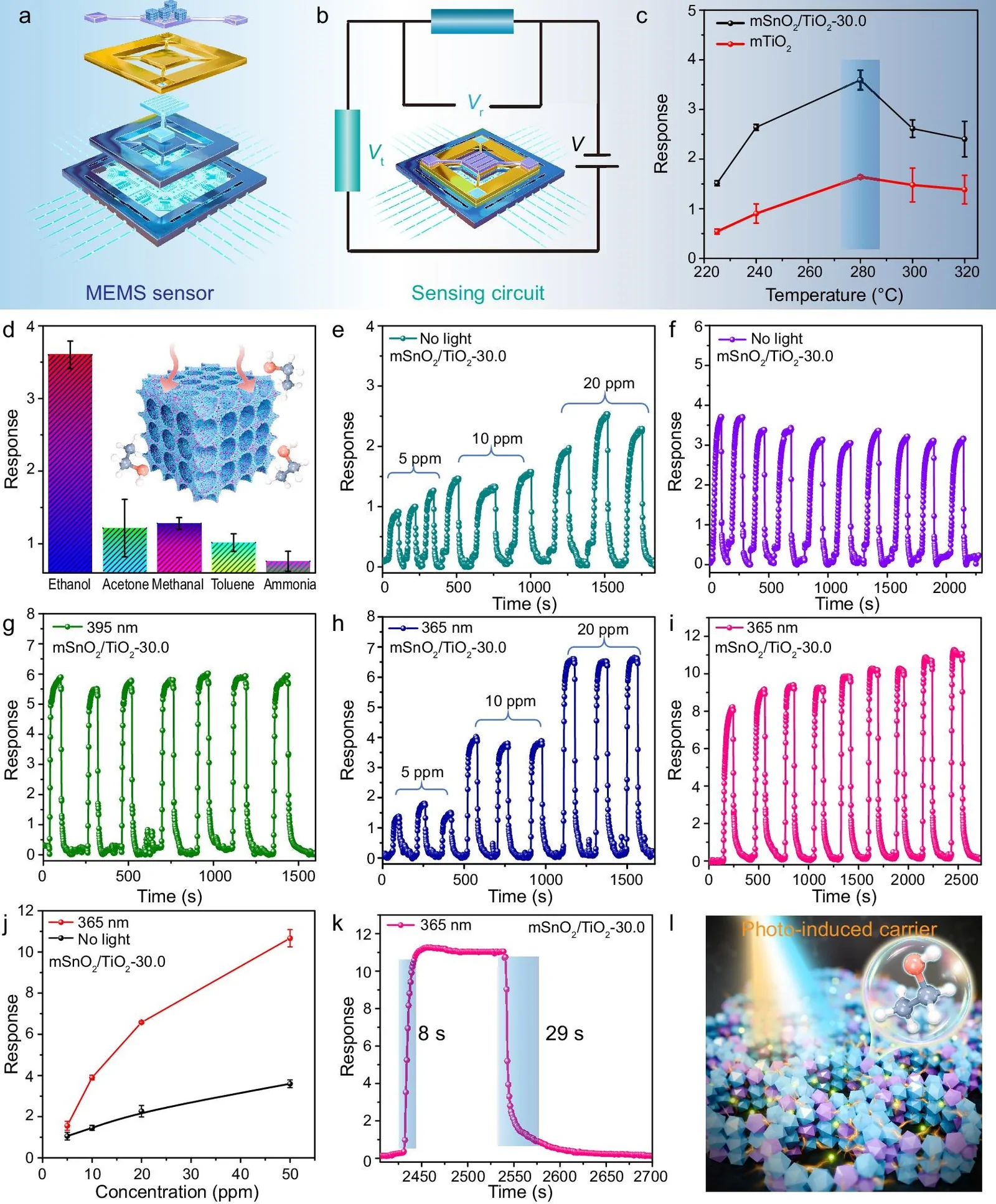
Study on the MEMS gas sensing performance of highly Sn^4+^-doped (5–30 wt.%) mSnO_2_/TiO_2_. (a) Schematic diagram of the MEMS sensor structure. (b) Sensing circuit detection principle. (c) Responses of the tailored sensors to 50 ppm ethanol without light at different working temperatures. (d) Selectivity testing toward 50 ppm gases and inserted schematic diagram of sensing behavior. Response–recovery curve of mSnO_2_/TiO_2_-30.0 toward ethanol of various concentrations (e) without light and (h) 365 nm light irradiation. Cycle stability test of mSnO_2_/TiO_2_-30.0 toward 50 ppm ethanol (f) without light, (g) 395 nm light irradiation and (i) 365 nm light irradiation. (j) Concentration-response correlation curves. (k) Dynamic response–recovery curve. (l) Photoassisted ethanol sensing mechanism.

To fully exploit the light-harvesting capability of hybrids, a UV LED lamp was employed as an auxiliary light source to investigate the ethanol sensing performance of mSnO_2_/TiO_2_-30.0 under illumination. The sensor exhibited enhanced response curves under 395 nm light irradiation condition, and its response to 50 ppm ethanol was about 6.0, which was two times higher than that without light (Fig. 6g). Similarly, it also showed typical response-concentration positive correlation and recovery cycle (Fig. 6h). Interestingly, the sensor showed self-enhancing sensing phenomenon toward 50 ppm ethanol under 365 nm light irradiation (Fig. 6i), and its response to 50 ppm ethanol was about 11.0, which was about four times higher than that without light conditions. It was ascribed to the light activation-induced effect. According to the concentration–response correlation curves (Fig. 6j), light irradiation significantly enhanced the MEMS sensor’s sensitivity to ethanol. It was mainly attributed to the material’s photoactivation effect and the role of photogenerated carriers in assisting the sensing process. For 50 ppm ethanol, mSnO_2_/TiO_2_-30.0 exhibited rapid response and recovery kinetics, with response and recovery times of 8 and 29 s, respectively (Fig. 6k). It is worth noting that the recovery process is relatively slow, and this is due to the formed heterojunction structure. Although it can enhance sensitivity, it increases interfacial barriers to photoexcited carrier separation and recombination, while gas adsorption and desorption-induced resistance changes require longer equilibration time. In addition, it indicated that the hybrid material facilitated the efficient spatial separation of photogenerated electron-hole pairs under photoexcitation, thereby improving charge carrier utilization, suppressing recombination and promoting the formation of surface-adsorbed oxygen species and reactive oxygen species. It facilitated the catalytic oxidation of ethanol molecules adsorbed on the material surface, and the hybrid material exhibited robust stability in ethanol sensing with/without light cycling conditions.

## CONCLUSIONS

In this study, tailored mesoporous SnO_2_/TiO_2_ composites with tunable pores structures, tailorable hetero-phase integration and engineered oxygen vacancies were synthesized via a facile ligand-mediated interfacial co-assembly strategy. p-hydroxybenzoic acid, as a bifunctional chelating ligand, selectively coordinates Sn^4+^ and Ti^4+^ precursors with disparate affinities, harmonizing their hydrolysis kinetics to achieve controlled Sn incorporation into the TiO_2_ framework. The ligand-mediated kinetic homogenization ensures uniform Sn distribution and fosters *in situ* crystallization of SnO_2_–TiO_2_ interfaces. The DFT(+*U*) modeling suggests that dilute Sn species mainly substitute TiO_2_ lattice sites with limited interstitial segregation, while elevated Sn contents drive the stabilization of well-defined heterojunctions. The optimized mSnO_2_/TiO_2_ delivers a photocurrent density of 25 μA cm^−2^ under neutral conditions and demonstrates robust, light-enhanced ethanol sensing on low-power MEMS platforms, highlighting its promise for photodriven sensors. This work advances a cut-and-sew architectural approach that orchestrates interfacial engineering and controlled assembly, offering an effective platform for photoactivated gas sensing.

## MATERIALS AND METHODS

### Materials

TBOT, PHBA, stannic chloride, monomethyl poly(ethylene oxide) (*M*_w_ = 5000 g mol^−1^), hydrochloric acid (35%–37% w/w) and acetic acid were purchased from Aladdin® Reagent Co., Ltd. THF and ethanol were obtained from Sinopharm Chemical Reagent Co., Ltd. All chemicals were used as received without further purification.

### Synthesis of tailored mesoporous SnO_2_/TiO_2_ composites

The amphiphilic block copolymer PEO_114_-*b*-PS_354_ (*M*_n_ = 41 874 g mol^−1^, PDI = 1.12) was designed and synthesized via the widely used atom transfer radical polymerization technique [49]. In a typical procedure, PEO_114_-*b*-PS_354_ (0.1 g) was dissolved in THF (6.0 mL) and stirred continuously for 15 min to yield a transparent solution, and then added 0.15 mL of concentrated hydrochloric acid (35%–37%) dropwise to the mixture and stirred for 2–3 min. Next, add 0.15 mL of acetic acid and continue stirring for 5–10 min. After the transparent solution is thoroughly mixed, add 0.3 mL of tetrabutyl titanate (TBOT) dropwise, and stir the mixture continuously for 0.5–1.0 h. When the solution turns pale yellow, 20 mg of PHBA is added rapidly, followed by a calculated amount of SnCl_4_ precursor. After further stirring for 0.5 h, a transparent solution is obtained, and the precursor solution is poured into a glass Petri dish and spread uniformly to enable co-assembly. It is subjected to stepwise thermal annealing to evaporate the solvent at 25°C for 24 h, then at 40°C for 24 h to completely remove residual solvent, and finally at 100°C for 24 h to induce structural fixation. The film obtained after annealing was scraped from the Petri dish and carbonized under a nitrogen atmosphere at 350°C for 3 h with a ramp of 1°C min^−1^, yielding tailored mesoporous hybrid materials with an sp^2^-hybridized carbon framework. Subsequently, the material was heated in a muffle furnace at 450 °C for 0.5 h with a ramp of 5°C min^−1^ to remove residual amorphous carbon, yielding the final highly crystalline hybrid metal oxide.

## Supplementary Material

nwag584_Supplemental_File
