# Supplementary material for: Interface engineering of mesoporous SnO2/TiO2 heterojunction with tailored defects for light-motivated gas sensor
Source: Natl Sci Rev. 2026 Sep 9;13(20):nwag584. doi: 10.1093/nsr/nwag584 (PMC13755761; doi:10.1093/nsr/nwag584)
Supplement: Supplementary file 1 [file nwag584_supplemental_file.pdf]

Supporting Information

Interface engineering of mesoporous SnO_2/TiO_2 heterojunction with tailored defects for light-motivated gas sensor

Yidong Zou[1,2], Yu Deng[2], Lingxiao Xue[2], Junhao Ma[2], Jichun Li[2], Tao Bo[4]*, Yonghui Deng[2]*, and Qin Yue[3]*

[1]School of Materials Science and Engineering, Tongji University, Shanghai 201804, P. R. China

[2]Department of Chemistry, State Key Laboratory of Coatings for Advanced Equipment, Shanghai Key Laboratory of Molecular Catalysis and Innovative Materials, iChEM, Fudan University, Shanghai 200433, P. R. China

[3]Institute of Fundamental and Frontier Sciences, University of Electronic Science and Technology of China, Chengdu 610054, P. R. China

[4]Ningbo Institute of Materials Technology and Engineering, Chinese Academy of Sciences, Ningbo 315201, P. R. China

*Corresponding authors.

E-mails: botao@nimte.ac.cn; yhdeng@fudan.edu.cn; qinyue@uestc.edu.cn

Characterization and testing

Small-angle X-ray scattering (SAXS) measurements were performed on the NanoStar U system (Bruker, Germany) using Cu Kα X-ray radiation at a tube voltage of 40 kV and a beam current of 35 mA. FESEM images were acquired using a Zeiss Ultra 55 field-emission scanning electron microscope (Carl Zeiss, Germany) at an accelerating voltage of 3.0 kV. TEM images were acquired using a JEOL JEM-2100F transmission electron microscope (JEOL, Japan) at an accelerating voltage of 200 kV. For FESEM analysis, samples were dispersed in ethanol and drop-cast onto silicon wafers, and the samples were dispersed in ethanol and loaded onto copper grids for TEM analysis. N_2 adsorption-desorption isotherms were measured using a Micromeritics ASAP 2420 surface area and porosity analyzer at 77 K, and the samples were degassed in vacuum at 180 °C for 6 h. The specific surface area was calculated by the BET method, and the pore size distribution was determined from the desorption branch of the N_2 adsorption/desorption isotherm using the BJH method. Total pore volume was estimated from the amount adsorbed at a relative pressure of P/P0 = 0.995. FT-IR spectra were recorded on a Nicolet FT-IR spectrometer using the KBr pellet method, and XPS spectra were acquired using a Shimadzu AXIS Ultra DLD spectrometer. Powder X-ray diffraction (XRD) patterns were recorded on a Bruker D4 ADVANCE diffractometer using Cu Kα radiation at 40 kV and 40 mA.

Photoelectric current test

The as-synthesized samples were fabricated as working electrodes for the systematic investigation of the photocurrent response of the $mSnO_2/TiO_2$ composites. Photocurrent measurements were performed using a CHI-660E electrochemical workstation under illumination from a 500 W xenon lamp. Meanwhile, the photocurrent performance of the hybrid materials was systematically compared: (i) across different Sn^{4+} doping concentrations, and (ii) in strongly alkaline (pH = 13.0), acidic (pH = 1.0), and neutral (pH = 7.0) electrolytes. Photoelectrochemical (PEC) characterization was performed in a conventional three-electrode electrochemical cell, including linear sweep voltammetry (LSV), chronoamperometric (CA) measurements under a fixed bias potential, and electrochemical impedance spectroscopy (EIS). The working electrode ink was prepared by dispersing 10 mg of the as-synthesized catalyst in 0.5 mL of ethanol, followed by the addition of 5 μL of Nafion® solution (5 wt.%, DuPont). The mixture was subjected to

ultrasonication for 30 min to yield a homogeneous catalyst ink, and then the slurry was drop-cast onto a 1.0 cm × 1.0 cm fluorine-doped tin oxide (FTO) glass substrate. The coated substrate was dried in an oven at 80 °C for 12 h to remove the solvent and ensure strong adhesion of the active layer, and a pure Pt mesh electrode and an Ag/AgCl electrode were used as the counter electrode and the reference electrode, respectively. LSV measurements were performed under illumination from a 500 W xenon lamp, scanning from -0.6 V to 1.8 V vs. Ag/AgCl at a scan rate of 50 $mV \cdot s^{-1}$. Transient photocurrent responses were recorded under illumination at a bias of 0.1 V vs. Ag/AgCl. EIS measurements were performed at 0 V vs. Ag/AgCl, over a frequency range of 1 Hz to 10^6 Hz, at room temperature (25 °C).

Gas sensing performance evaluation

Gas sensing measurements were performed using the MEMS chips, and the sensing films were fabricated via drip-coating, followed by drying and annealing. First, the powdered hybrid material was dispersed in deionized water to form uniform slurry of 10 mg/mL by ultrasonic treatment for 20-30 min. The slurry was drop-coated onto the electrode region of MEMS chips and then drying, repeating the coating process 2-4 times. The sensing response is defined as $S = |V_a/V_g|-1.0$, where Va is the voltage of the sensor in air and Vg is its voltage in the target gas. Furthermore, the response time is defined as the time required for the sensor voltage to reach 90% of its response peak upon exposure to the target gas, while the recovery time is the time required for the voltage to recover to 90% of its baseline value (V_a) in air. All tests were conducted at relative humidity levels below 60% RH, and each measurement was repeated 3-5 times. The average measurement error was approximately 5%.

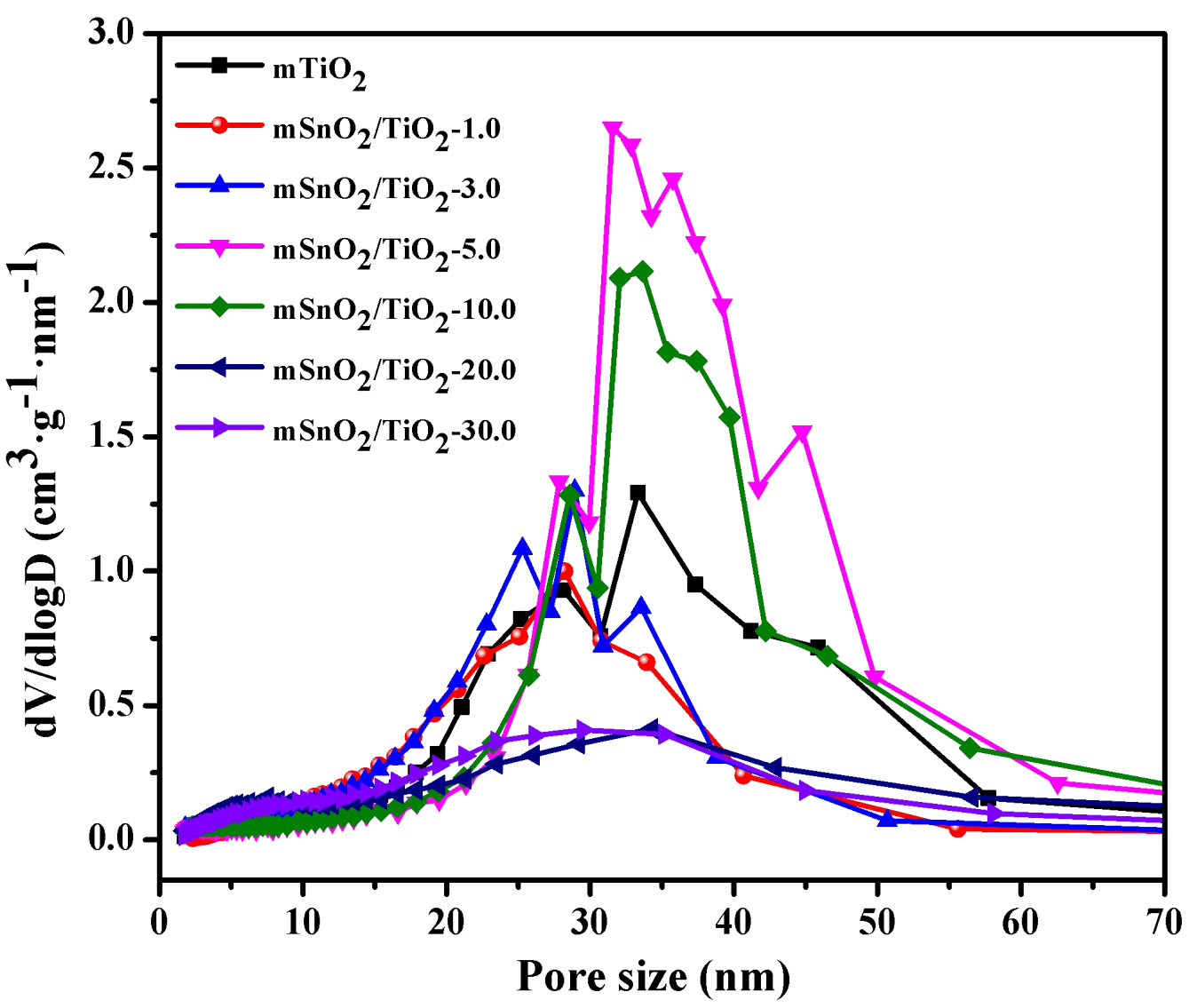

Figure S1. The corresponding pore size distribution curve of the crystalline $mSnO_2/TiO_2$-*X* (*X* = 1.0%, 3.0%, 5.0%, 10.0%, 20.0%, and 30.0%).

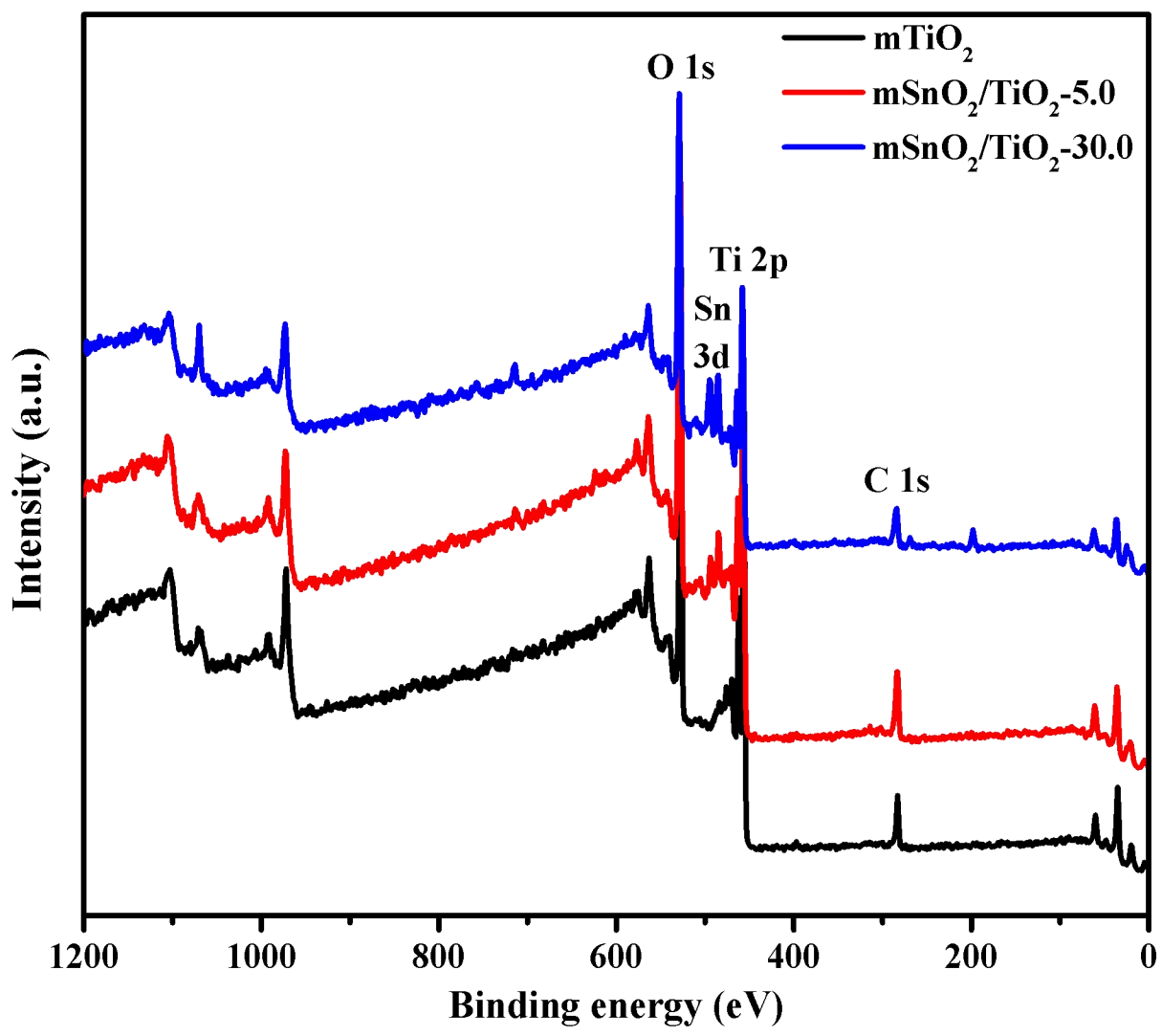

Figure S2. XPS spectra of $mTiO_2$, $mSnO_2/TiO_2$-5.0 and $mSnO_2/TiO_2$-30.0 composites.

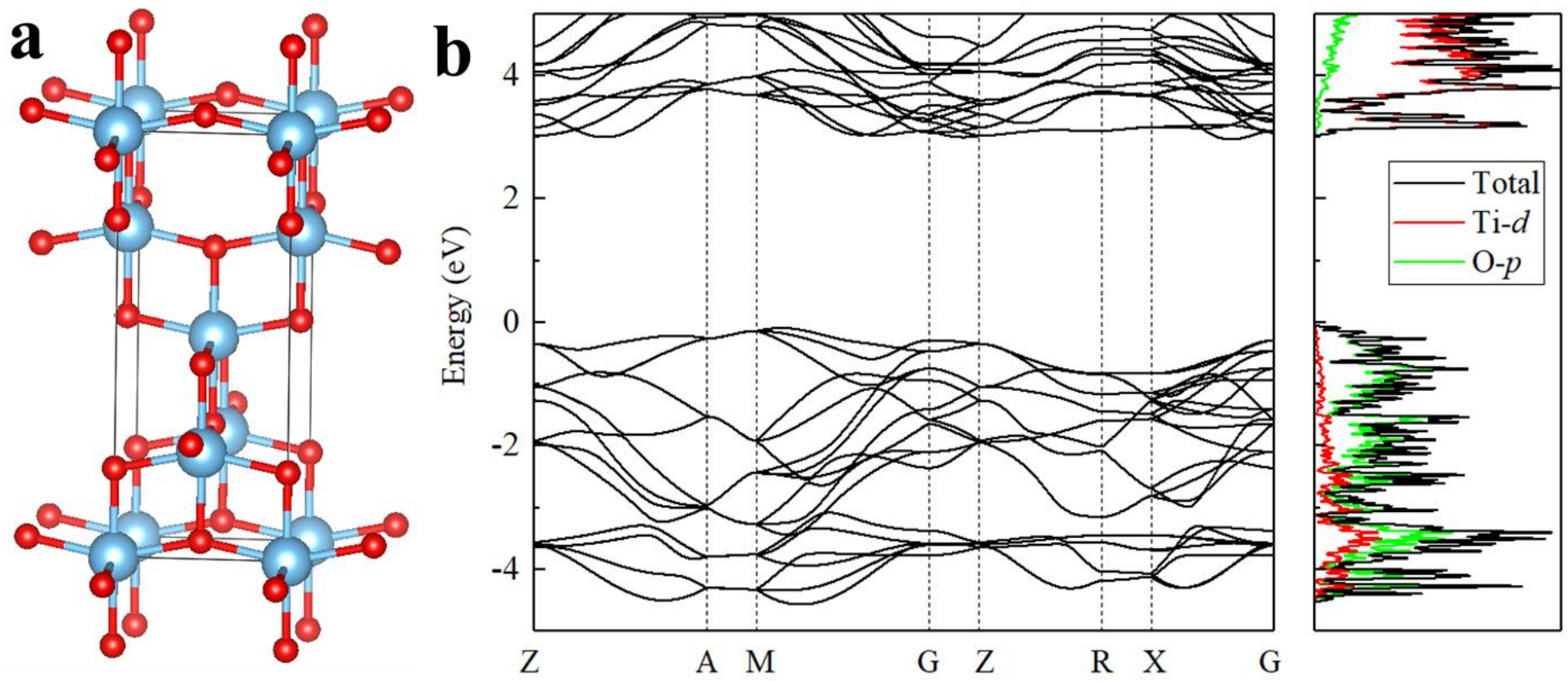

Figure S3. (a) The optimal TiO_2 unit cell structure (red: oxygen atoms; blue: Ti atoms), (b) The TiO_2 band structure with U = 8.5 eV.

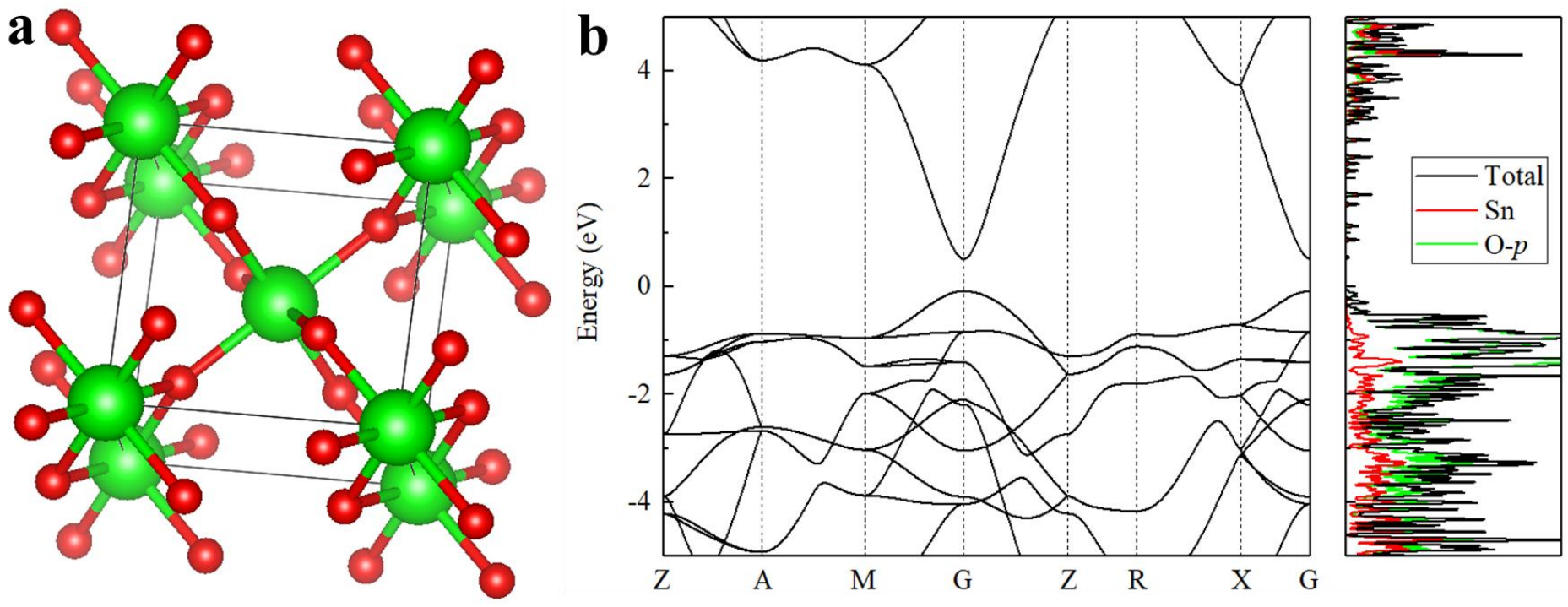

Figure S4. (a) The optimal SnO_2 unit cell structure (red: oxygen atoms; green: Sn atoms), (b) The energy band structure of SnO_2.

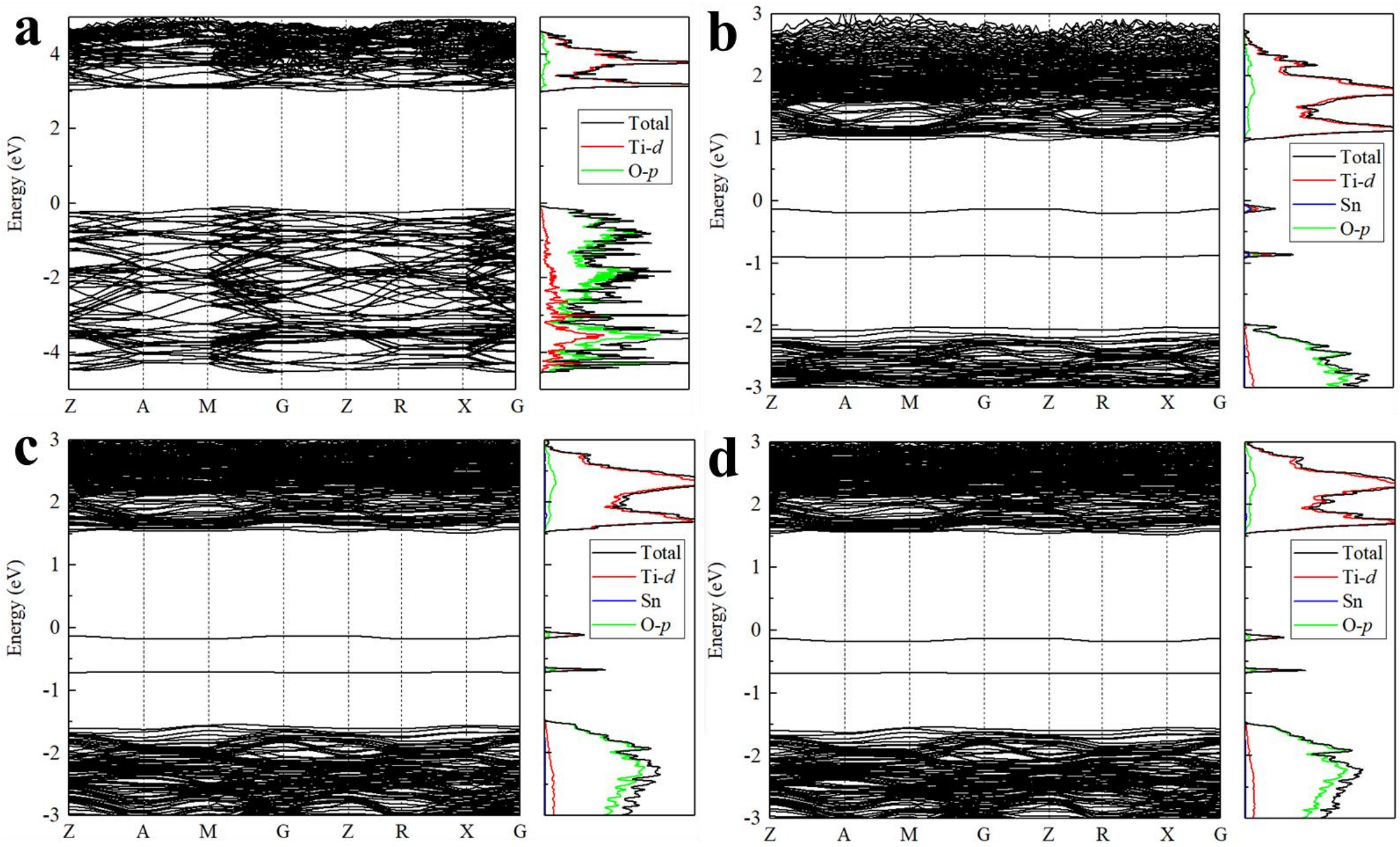

Figure S5. (a) Band structure of 3*3*1 unit cell TiO_2, (b) Band structure of Sn doping at the interstitial lattice, (c) Band structure of Sn replaces Ti atom (type-I), (d) Band structure of Sn replaces Ti atom (type-II).

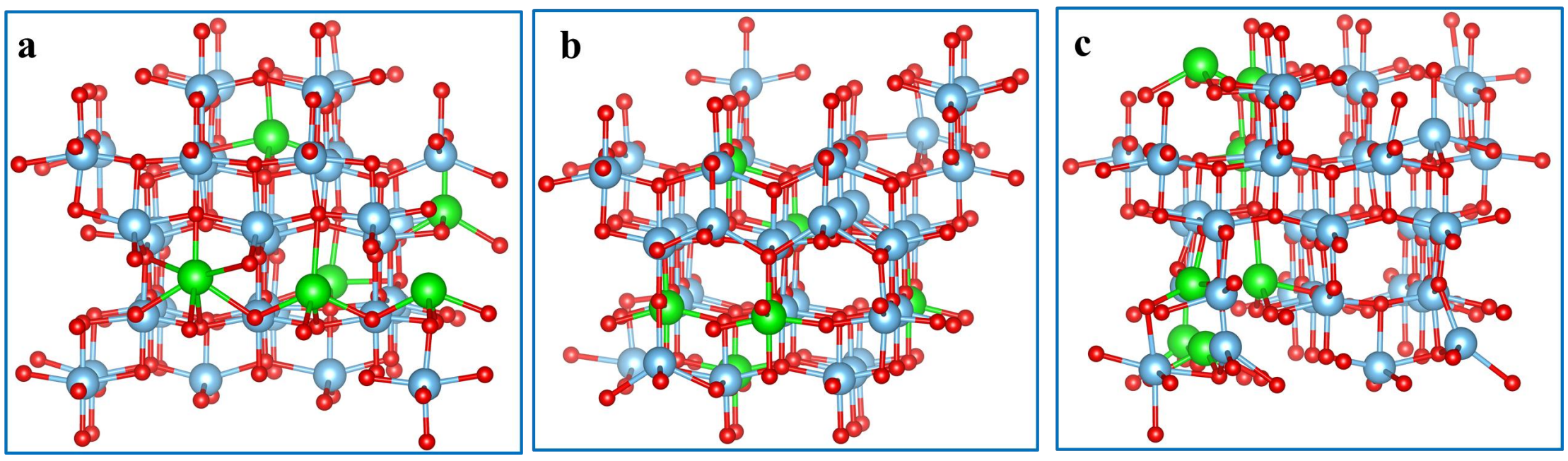

Figure S6. The Sn^{4+} doped SnO_2/TiO_2 hybrid structure (non-heterojunction structure) with a doping amount of 30.0 wt.% (red: oxygen atoms; blue: Ti atoms; green: Sn atoms): (a) Hybrid structure of Sn fully doped in the interstitial lattice, (b) Hybrid structure of Sn completely replaces Ti, (c) Hybrid structure of Sn fills the gaps and replaces Ti in half.

Table S1. The pore size structural parameters of $mSnO_2/TiO_2$-X (X = 0-30 wt.%) hybrid materials.

**Samples**	**Pore size D (nm)** [a]	S_{BET}[b] **($m^2 \cdot g^{-1}$)**	V_t[c] **($cm^3 \cdot g^{-1}$)**
$mTiO_2$	33.3	93.64	0.46
$mSnO_2/TiO_2$-1.0	28.2	94.11	0.36
$mSnO_2/TiO_2$-3.0	28.9	106.72	0.40
$mSnO_2/TiO_2$-5.0	31.6	116.71	0.64
$mSnO_2/TiO_2$-10.0	33.6	111.86	0.57
$mSnO_2/TiO_2$-20.0	34.3	97.94	0.31
$mSnO_2/TiO_2$-30.0	29.5	85.33	0.29

[a]*D*: Pore size calculated by the BJH model; [b]S_{BET}: Specific surface area calculated by the BET method; [c]V_t: Total pore volume.

Table S2. Relative contents of various oxygen species of $mTiO_2$, $mSnO_2/TiO_2$-5.0 and $mSnO_2/TiO_2$-30.0 calculated from XPS analysis

Samples	O 1s		
	Lattice oxygen (Ti-O or Ti-O-Sn)	Defective oxygen (O_v)	Adsorbed molecular oxygen (O_{abs})
$mTiO_2$	51.2%	25.5%	23.3%
$mSnO_2/TiO_2$-5.0	81.3%	9.9%	8.8%
$mSnO_2/TiO_2$-30.0	31.6%	51.2%	17.1%

Table S3. Optimal band results and related parameters of TiO_2 unit cell structure under different U value optimization in the optimal state.

**U (eV)**	**3**	**4**	**5**	**6**	**7**	**8**	**8.5**	**9**	**9.5**	**10**
Conduction band bottom	1.987	2.217	2.364	2.609	2.766	2.945	3.001	2.890	2.889	2.767
Value band edge	-0.186	-0.119	-0.144	-0.083	-0.108	-0.116	-0.088	-0.150	-0.102	-0.174
Band gap	2.173	2.336	2.508	2.692	2.874	3.061	3.089	3.040	2.991	2.941

Table S4. DFT calculation parameters of the SnO_2/TiO_2 hybrid material with a doping content of 30%.

Binding energy (eV)	Sn fully doped in the interstices	All Sn replaces Ti	Sn filled in half of the space and replaced Ti in the other half
Structure 1	-708.846	-710.968	-710.416
Structure 2	-707.590	-711.273	-709.887
Structure 3	-708.028	-711.166	-710.508
Structure 4	-709.398	-711.855	-709.491
Structure 5	-709.374	-710.522	-709.814
